# Discrepancies between the reported item counts and the appendices of the Neurocore-SENSED consensus^☆^

**DOI:** 10.1016/j.bas.2026.107477

**Published:** 2026-10-15

**Authors:** Abdullah İyigün

**Affiliations:** University of Health Sciences, Istanbul Ümraniye Health Research Center, Department of Orthopaedics and Traumatology, Istanbul, Türkiye

Dear Editor,

I read with interest the article by Al Barajraji et al. reporting the Neurocore-SENSED consensus, which establishes a core outcome set (COS) and reporting guidelines for endoscopic spine surgery (ESS) in disc disease (Al Barajraji et al., 2025). The authors are to be commended for assembling a genuinely international and decentralized panel and for addressing a heterogeneity problem that has long impeded comparative research in this field. Because a COS is intended to be adopted, audited, and reused by other investigators, the item-level data that underpin it must be fully reconstructable from the published record (Kirkham et al., 2017). While mapping the published items onto a protocol for an ESS cohort at our institution, I encountered two arithmetic inconsistencies between the appendices and the corresponding text and tables. I raise them here that they can be resolved, since the framework itself is a valuable contribution.

## 1 Appendix C contains fewer items than the stated total for the practices domain

Appendix A specifies 86 items for the practices domain (clinical 21, surgical 37, technical 28), a figure reproduced in Table 1 and in section 3.4. Direct enumeration of Appendix C, however, yields 69 items (clinical 18, surgical 28, technical 23), a shortfall of 17.

**Table 1:** Practices domain: counts reported in the article versus counts enumerated from Appendix C.

Agreement category	Original article	Appendix C (enumerated)	Difference
Strong agreement (≥75 %)	32	32	0
Moderate agreement (66–74 %)	7	7	0
No agreement (<66 %)	47	30	−17
**Total items**	**86**	**69**	**−17**

The shortfall falls entirely within a single agreement category. Counting the agreement labels printed in Appendix C reproduces Table 1 exactly for strong agreement (n = 32) and for moderate agreement (n = 7), whereas the no-agreement count derived from Appendix C is 30, against the 47 reported in Table 1 of the article (Table 1 below).

That every missing item belongs to the non-consensus category, while every consensus item is intact, suggests a row-loss or typesetting error during production of the appendix rather than an error in the underlying analysis. The omission is nonetheless consequential. In their absence, readers cannot determine which technical or surgical parameters were deliberated and rejected, as opposed to never having been proposed.

## 2 The moderate and no-agreement counts in the patient characteristics domain differ by one item

Appendix B lists the full complement of 65 patient characteristics items, matching Appendix A for every subdomain (demographics 13, comorbidities 11, initial complaints 8, severity assessment 22, ongoing treatments 11), and the strong-agreement count derived from it (n = 30) again matches Table 1. The distribution of the remaining items, however, does not reconcile (Table 2).

**Table 2:** Patient characteristics domain: counts reported in the article versus counts enumerated from Appendix B.

Agreement category	Original article	Appendix B (enumerated)	Difference
Strong agreement (≥75 %)	30	30	0
Moderate agreement (66–74 %)	8	7	−1
No agreement (<66 %)	27	28	+1
**Total items**	**65**	**65**	**0**

Only seven items in Appendix B carry a moderate-agreement label: tobacco consumption (74 %), active infections (71 %), cardiovascular diseases (66 %), osteoporosis (66 %), acetaminophen (74 %), pain modulation implantable devices (69 %), and muscle relaxants (67 %). No further item falls within the 66–74 % band defined in section 2.3, and no item within that band is labeled otherwise, so the eighth moderate item reported in Table 1 and section 3.3 cannot be identified from the published data.

This single-item discrepancy propagates to the principal numerical finding of the study. Summing the agreement labels across Appendices B, C, and D yields 82 items with strong agreement and 16 with moderate agreement, that is, 98 items reaching consensus, rather than the 99 items (82 strong, 17 moderate) reported in the Abstract and in section 4. The outcomes domain (Appendix D) is internally consistent throughout: 17 clinical and 15 complication items, distributed as 20 strong, 2 moderate, and 10 without agreement, in exact agreement with Tables 1 and 4.

## 3 Request

I would be grateful if the authors could clarify the discrepant counts and, ideally, publish complete item-level appendices as supplementary material, including every item that failed to reach consensus together with its selection rate. A corrigendum of this nature would cost the authors little and would allow the Neurocore-SENSED framework to function fully as the reference standard it was designed to be. None of the observations above detracts from the methodological quality or the value of this consensus; they concern the completeness of its published record, which for a core outcome set is inseparable from its utility.

## Funding

This work received no funding.

## Conflicts of interest

The author declares no competing financial or personal interests.

## References

[bib1] Al Barajraji M., Barrit S., Boukind A. (2025). Spine endoscopic surgery establishment for disc disease (Neurocore-SENSED): an open and decentralized consensus. Brain Spine.

[bib2] Kirkham J.J., Davis K., Altman D.G. (2017). Core outcome Set-STAndards for development: the COS-STAD recommendations. PLoS Med..

